# Improving the ischemia-reperfusion injury in vascularized composite allotransplantation: Clinical experience and experimental implications

**DOI:** 10.3389/fimmu.2022.998952

**Published:** 2022-09-16

**Authors:** Jiqiang He, Umar Zeb Khan, Liming Qing, Panfeng Wu, Juyu Tang

**Affiliations:** ^1^ Department of Hand and Microsurgery, Xiangya Hospital of Central South University, Changsha, China; ^2^ National Clinical Research Center for Geriatric Disorders, Xiangya Hospital of Central South University, Changsha, China

**Keywords:** ischemia-reperfusion injury (IRI), vascularized composite allotransplantation (VCA), tissue damage, transplant rejection, innate immunity, adaptive immunity

## Abstract

Long-time ischemia worsening transplant outcomes in vascularized composite allotransplantation (VCA) is often neglected. Ischemia-reperfusion injury (IRI) is an inevitable event that follows reperfusion after a period of cold static storage. The pathophysiological mechanism activates local inflammation, which is a barrier to allograft long-term immune tolerance. The previous publications have not clearly described the relationship between the tissue damage and ischemia time, nor the rejection grade. In this review, we found that the rejection episodes and rejection grade are usually related to the ischemia time, both in clinical and experimental aspects. Moreover, we summarized the potential therapeutic measures to mitigate the ischemia-reperfusion injury. Compare to static preservation, machine perfusion is a promising method that can keep VCA tissue viability and extend preservation time, which is especially beneficial for the expansion of the donor pool and better MHC-matching.

## Introduction

The world of reconstructive transplantation is mature (1). The challenges of allograft rejection have focused research on the long-term success of vascularized allograft transplantation (2, 3). Ischemia-reperfusion injury (IRI) is a potential threat to long-term allograft success, which is an inevitable event that follows reperfusion after a period of cold static storage (4, 5). This review summarizes the current clinical and laboratory aspects that discuss the relationship between transplant outcomes and IRI tissue damage. It can give some implications to reduce the IRI to achieve long-term VCA allograft survival.

## Mechanisms of IRI

Ischemia leads to hypoxic anaerobic glycolysis and oxygen consumption, depleting adenosine triphosphate (ATP) and dysregulating ATP-dependent membrane ion exchangers (6, 7), reducing the activity of the Na+/K+/ATPase pump and increasing intracellular sodium concentration (8). Furthermore, the reduction in the intracellular concentration of ATP prevents the regeneration of glutathione, ascorbic acid and tocopherol that take part in detoxifying the metabolites present in the cytosol and the sarcoplasmic membrane. The accumulation of osmotically active particles such as lactate, sodium, inorganic phosphate and creatine leads to cell edema.

Moreover, cellular acidosis can stimulate the antiport Na+/H+ receptors, worsening the sodium overload and affecting the function of other membrane receptors such as the Na+/Ca2+ antiport. The Na+/Ca2+ antiport enables sodium exportation from cells based on the intracellular calcium concentration (9). Cellular hypercalcemia causes the breakdown of sarcoplasmic phospholipids and cytoskeleton protein, alters contractile protein’s efficiency and calcium affinity, and changes the tertiary structure of certain enzymes such as xanthine dehydrogenase to xanthine oxidase (10). These two enzymes have similar functions: the transformation of hypoxanthine in xanthine and xanthine in uric acid. Damage to calcium-dependent receptors increases cytosolic calcium, loss of homeostasis, activation of proteolytic enzymes, cell membrane disruption, and release of free fatty acids. Collectively, this dysfunction manifests as cell apoptosis or necrosis (11–13).

Reperfusion triggers a localized microvascular and systemic reaction, resulting in further tissue damage (14–16). Mitochondria respond to many different types of stress like oxidative and metabolic stresses (17–19). They are the primary source of reactive oxygen species (ROS), a by-product of respiration generated mainly at the electron transport chain complexes I and III (20). Ca2+ overload, with high ROS and Pi, changes mitochondrial membrane permeability and induces the opening of non-selective and high-conductance permeability transition pores (PTP) in the inner mitochondrial membrane (21–23). The PTP further compromises mitochondria’s bioenergetics function and structural integrity, leading to cell death (24–26). The release of ROS, mainly from mitochondria, forms the basis for IRI (27, 28).

## How the IRI influences the transplant outcome

### Experimental implications

#### IRI in the skin and subcutaneous tissue

IRI in the skin has been reported in several publications, not only in VCA but also in flap surgery (29–31). Skin and subcutaneous tissue are relatively resistant to the effects of anoxia, and intracellular pH changes are reversible for up to 24 h (32). Donski et al. (33) investigated the effect of cooling on the survival of free flaps in rabbit. They found 86% of flaps that were cooled for 1–3d survived. Meanwhile, other authors concluded that the maximum ischemia time of a rat flap was 6h at normal body temperatures and 48h if cooled (34). Thus, the warm IRI has more serious tissue damage than the cold IRI.

As VCA tissue is usually preserved at 4 °Cfor 6h, the warm ischemia time in VCA skin and subcutaneous tissue is pretty short. The tissue damage in the skin and subcutaneous tissue can be ignored. However, the IRI should be considered if warm ischemia is >6h or cold ischemia time >24h.

#### IRI in the skeleton muscle

Compared to the skin, mammalian skeletal muscle is substantially less tolerant to ischemia (35). Irreversible damage to the microcirculation of skeletal muscle in man begins at around 6 h (36). Wagh et al. (37, 38) found that skeletal muscle is much more susceptible to damage from cold (4°C) ischemic storage than skin, with an estimated critical ischemia time for rat gastrocnemius muscle flaps of approximately 16 h compared with approximately 3.5 days for rat epigastric skin flaps. Although measures have been taken to ameliorate the IRI in muscles, lots of results are based on short time warm-ischemia time (39–43). The data for VCA clinical usage is limited.

#### IRI in the vessel

The endothelium is very sensitive to I/R injuries (44, 45). It is essential to preserve the endothelium because endothelial cells have several vital functions, including controlling vascular tone and local blood flow, modulating coagulation and inflammation, participating in immune response, regulating micro and macromolecules’ movement towards the interstitium, and assisting in angiogenesis (46). Endothelium-dependent vasodilatation is more susceptible to IRI than vasoconstriction and endothelial-independent vasodilatation (47, 48). ROS and tumor necrosis factor-alpha(TNF-α) play a significant role in this process. Reperfusion also induces a critical inflammatory response, characterized by a massive production of free radicals and activation of the complement pathway, leucocytes and neutrophils (49). A little interaction between activated endothelium and neutrophils will result in a significant concentration of activated neutrophils in the interstitium, which release oxygen radicals and proteases, leading to the destruction of cells and the extracellular matrix. The migration of neutrophils from the intravascular bed to the interstitium involves several families of proteins such as selectins (P-selectin and L-selectin), integrins (intercellular adhesion molecule-1), and immunoglobulins (platelet-endothelial cell adhesion molecule-1). Lastly, oxidative stress, cytokine production, and the secondary mitochondrial lesions that occur with reperfusion induce apoptosis in parenchymal cells and the vascular structures.

In addition, vascular endothelial cells are the initial barrier to allograft-activated host immune rejection and are critical in triggering cell-mediated acute rejection (50). It has been found that circulating mitochondria in organ donors with prolonged ischemia may directly activate allograft vascular endothelial cells and promote graft rejection (51–53). Therefore, endothelial cells mediate acute graft rejection after IRI. The targeted intervention of mitochondrial damage in vascular endothelial cells, thereby reducing graft rejection events, has also been a research hot-spot in recent years.

#### IRI in the nerve

Although much is known, the precise pathophysiology of IRI in the peripheral nerve remains to be elucidated. Microvascular events, which may occur during reperfusion, may be important in amplifying the nerve fiber degeneration that is initiated during ischemia (54). Haruyasu Iida et al. (55) showed that reperfusion induced oxidative damage, which lowered nerve function and increased fiber deterioration, but extending the period of reperfusion to 42 days allowed for fiber regeneration. To reduce oxidative injury, Sang-Jin Shin et al. (56) investigated how inducible nitric oxide synthase (iNOS) inhibition affects the recovery of motor function in the rat sciatic nerve after IRI. Their study indicated that early inhibition of iNOS is vital for IRI reduction or prevention. Franka et al. (57) studied the critical ischemia times of individual tissues of a rat limb isograft. Histomorphometric investigation of the tibial nerve on POD 10 showed the typical signs of Wallerian degeneration in all transplanted animals and the nerve transection groups. The nerve of non-transplanted controls appeared to be normal in shape without signs of injury or cell infiltration. Overall, histopathological scores for nerve damage were significantly higher in the ischemia group than transection group. In general, nerve scores increased proportionally with the duration of ischemia time.

#### IRI in the bone

Compared to most other organs, the bone’s IRI is poorly understood, particularly from a mechanistic perspective. However, IRI of the bone is considered to occur in various diseases/situations (58–61). such as vascular disruption or compression, fractures, limb replantation/allotransplantation, and thromboembolic disorders. Moreover, some systemic diseases such as sickle cell anemia, Caisson disease, and Cushing’s disease may initiate IRI in the bone (62–64).

As systemic diseases influence many organs, it’s hard to investigate the mechanism of reperfusion injury in bones. Thus, bone IRI has been studied by interrupting blood supply through vascular compression (clamping) or dissection. In these studies, limb or bone graft replantation/transplantation was performed after preserving the limbs/grafts at 0 – 4 °C or room temperature (21 –25°C) (65–68). The studies concluded that significant retardation of bone growth/development occurs when critical ischemia lasts between 3 to 7 h at 37 °C. But the critical ischemia time increases with decreasing temperature. In some studies, even cold ischemia time (0 – 5 °C) of 25 h and above have been found to be tolerated (69).

The fact that therapy with antioxidants resulted in considerable protection proves that reperfusion injury of the bone, or extra injury during the reperfusion period, occurs (70). This reiterates the protective function of antioxidants against ROS. ROS can only be formed in the presence of O2, which means upon reperfusion. The available results, however, suggest some similarities to the mechanisms of IRI of other organs, such as the involvement of ROS (71, 72).

From a review of the literature, we have summarized the following critical ischemia time of VCA tissues (**Table 1**):

**Table 1:** Critical ischemia time of VCA tissues.

Tissue	Warm	Cold
skin and subcutaneous tissue	4–6h	up to 12h
muscle	<2h	8h
nerve	8h	24h
vessel	6h	12h
bone	<3h	24h

Many papers reported the IRI tissue damage in VCA. We briefly introduce the tissue damage in different types of VCA tissue. The lack of blood supply does not damage all tissues in the allografts to the same degree; some tissues are more susceptible than others. Those damaged tissues may release some molecules and activate the innate immune response, which is a barrier to long-term allograft survival (5, 73–75). In this review, we are not only focused on the relationship between the IRI and tissue damage but also discuss the relationship between IRI and transplant rejection caused by tissue damage (**Table 2**).

**Table 2:** Relationship between IRI and tissue damage or transplant rejection.

Year	Author	Species	Model	Ischemia time	Preservation solution	Follow up	Conclusion
2009	Pradka, S. P (76)	Rat	Allogeneic vascularized epigastric flaps	1h or 3 h WI	Heparinized saline solution	POD 6	Skin and muscle demonstrated increased acute rejection of allotransplants with increased subcritical ischemic time
2010	Xiao, B (77)	Rat	Allogeneic vascularized groin flaps	0h, 6h, 12h, 18h, or 24 h CI	University of Wisconsin	POD 2-8	Prolonged ischemia has a deleterious effect on allograft survival
2010	Fumiaki Shimizu (78)	Rat	Allogeneic vascularized groin flaps	1h or 6 h WI	N/A	POD 14	Longer ischemic time induces more severe rejection against allo-transplanted tissue compared with the shorter one
2012	Villamaria, C. Y (79)	Swine	Gracilis musculocutaneous flap	1 h CI or 3 h CI	Heparinized saline solution	POD 1 to POD14	Skeletal muscle tissue injury (LDH, CK, and AST) showed ischemia period-dependent response
2014	Hautz (80)	Rat	Syngeneic hindlimb transplantation	2 h CI or 10 h CI, or 30 h CI	Saline or Histidine-tryptophan-ketoglutarate, or University of Wisconsin	POD 10	Severe inflammation and tissue damage are observed after prolonged cold ischemia in muscle and nerve
2016	Bonastre, J (81)	Rat	Allogeneic orthotopic hindlimb transplantation model	7h CI	Heparinized saline solution	2 months	An association between cold ischemia and chronic rejection was observed in experimental vascularized composite allotransplantation
2017	Datta, N (82)	Mouse	Allogeneic orthotopic hindlimb transplantation model	1h h CI or 6 h CI	University of Wisconsin	POD 1 to POD 3	Prolonged cold ischemia triggers progressive IRI with vascular endothelial damage
2017	Messner, F (57)	Rat	Syngeneic hindlimb transplantation	2 h CI or 6 h CI, or 10 h CI	Saline or Histidine-tryptophan-ketoglutarate, or HTK-N, or TiProtec	POD 10	Muscle and nerve injury was significantly aggravated after prolonged cold ischemia
2017	Fries, C. A (83)	Swine	Gracilis musculocutaneous flap	3h CI	Heparinized saline solution or C1 esterase inhibitor	POD 1 to POD14	C1inhibitor is protective of IRI and may have utility in vascularized composite allotransplantation
2018	Robbins, N (84)	Swine	Heterotopic myocutaneous flap(autotransplants and allotransplants)	5 h CI or 14 h CI, 17h machine perfusion	University of Wisconsin	14 days for autotransplants and 60 days for allotransplants	Machine perfusion protecte ischemic damage and chronic rejection following allotransplantation in the porcine model
2020	Gok, E (85)	Rat	Syngeneic hindlimb transplantation	6h h WI or 6 h CI	Histidine-tryptophan-ketoglutarate	12 weeks	Limb allografts suffer from irreversible muscle damage without circulation by 4 h and have functional deficits on cold ischemia at 6 h

IRI, ischemia-reperfusion injury; h, hours; CI, cold ischemia; WI, warm ischemia; POD, postoperative day; N/A, not available.

### Clinical experience

Ischemia is clinically an inevitable factor following donor organ procurement, cold preservation, and implantation. Though its specific role in VCA is occasionally underappreciated, the IRI can affect graft survival, function, and rejection. However, there is a paucity of studies examining IRI in VCA clinical usage. The experience in re-transplantation has opened a window for us to know the critical ischemia time related to VCA. The recommended ischemia times compatible with reliable success in replantation are 6 h of warm and 12 h of cold ischemia for major replants, although successful replantations have been reported after longer ischemia times (86–89). The ischemia time is largely influenced by skeleton muscle and causes it even more susceptible to IRI. Besides tissue damage, the literature in SOT has clearly demonstrated that IRI is a potent activator of the immune system and subsequently leads to occur rejection episodes. These clinical findings have also been testified in VCA. Based on the presence of a more aggressive diffuse lymphocytic infiltration and disruption of tissue architecture, Pradka et al. (90) showed that skin and muscle exposed to 3 h of ischemia had significantly higher rejection scores than when exposed to 1 h of ischemia.

To sum up, the IRI in VCA has the following effect on the graft and body.

#### Graft survival

Insult to the endothelium leads to an upregulation in the expression of bioactive agents (such as endothelin and thromboxane) and suppresses the release of nitric oxide (NO), increasing vascular tone. The presence of free radical accumulation, endothelial edema, and platelet activation can result in the cessation of blood flow through the graft. Thus, graft survival is threatened by IRI (91, 92).

#### Tissue damage

Muscle and nerve dysfunction in the donor limb has a significant impact even if the graft survives since their physiological integrity is crucial for maintaning physical function. We clearly described the IRI tissue damage in experimental implications. The muscle tissue is sensitive to the IRI, then the skin and subcutaneous, bone is more tolerant to IRI (93–95). However, the muscle normal structure and functional recovery are very important to the VCA surgical outcomes. Besides, the neuromuscular junctions are most sensitive to ischemia (96). Since most organs are still preserved in static preservation, muscle tissue is more sensitive to IRI than other organs, and it is not easy to regenerate after IRI, reducing IRI tissue damage has a positive effect on the functional recovery of VCA.

#### Graft rejection

**Figure 1:**
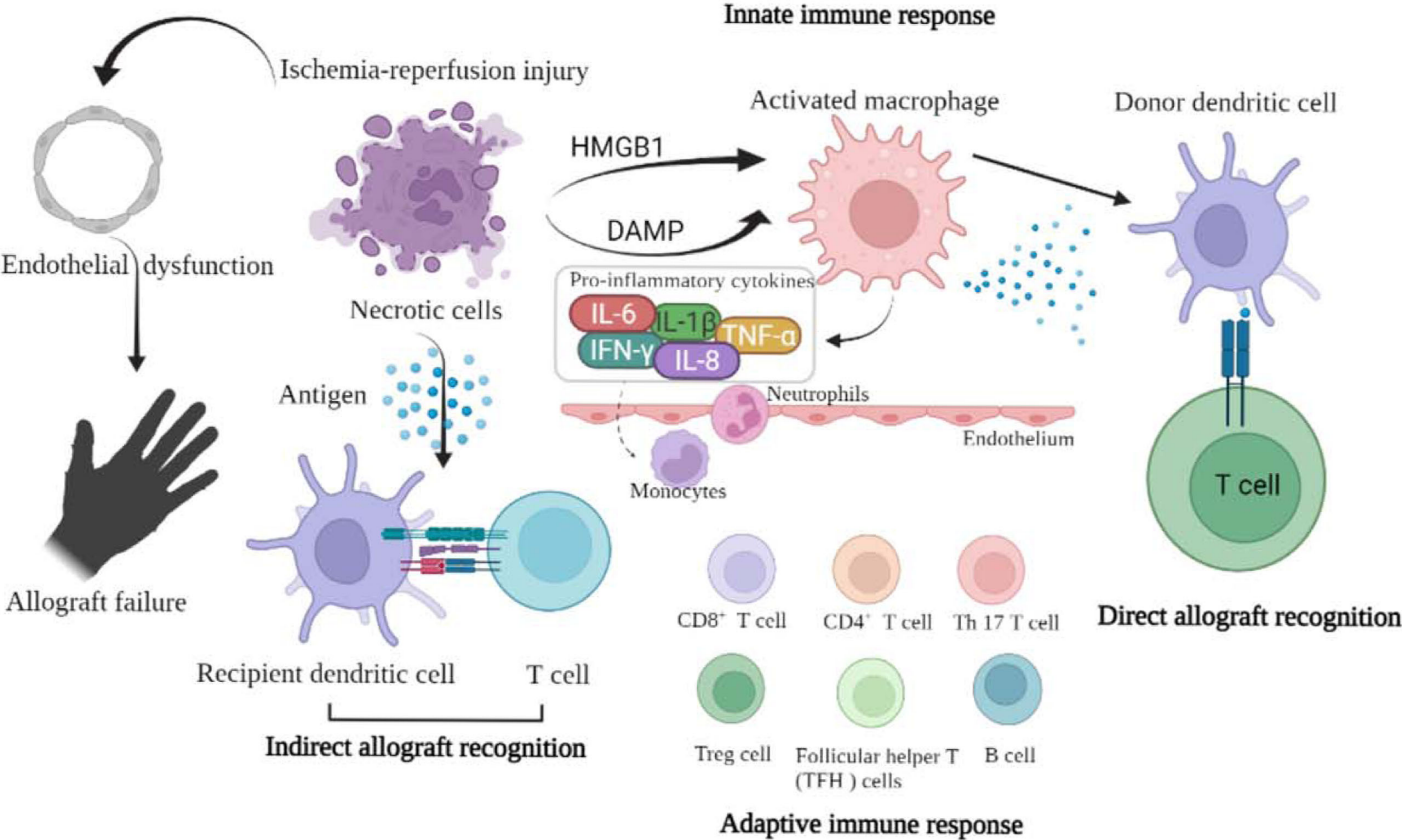
The schematic picture shows the relationship between ischemia-reperfusion injury and graft rejection.

As it is well known, there are 3 necessary steps for graft rejection. Step 1: Leukocyte infiltration (endothelial cells dysfunction, P-selectin and L-selectin, intercellular adhesion molecule-1, platelet-endothelial cell adhesion molecule-1). Step 2: Leukocyte activation (local inflammation). Step 3: Target organ (effective T cells and cytokines producing). Injured tissues activate the immune system by releasing damage-associated molecular patterns, which are endogenous molecules that mediate cellular injury. Their release upregulates endothelial adhesion molecules, migration of leukocytes into the graft and complement activation. This localized inflammatory response hampers the development of tolerance (97, 98). This explains why post-transplant graft dysfunction and rejection are proportional to the intensity and duration of IRI (**Figure 1**). Thus, ameliorating the IRI in VCA may reduce the incidence of VCA rejection (99).

#### Systemic reperfusion injury

Reperfusion injury may lead to systemic metabolic changes and the release of oxidized free radicals in patients, leading to cellular oxidative stress, systemic inflammatory response, multiple organ failure, and eventually death. Based on current arm replantation experiences, there is a chance of local or systemic complications, such as sepsis, remote organ failure, hyperkalemia, or acidaemia (100–104). Thus, the systemic reperfusion injury should be seriously evaluated prior to VCA surgery (105).

## Strategies to reduce IRI

Over the past 20 years, a variety of drugs and interventions have been reported in clinical and basic research to alleviate IRI. Many treatment methods are based on limb IRI models, limb autograft models, and limb replantation. These interventions have shown good therapeutic effects, and the research results are worthy of reference by VCA. **Table 3** summarizes the most commonly used treatments to reduce IRI, which include adenosine agonists, endothelin antagonists, antioxidants, complement activation inhibitors, apoptosis inhibitors, anti-inflammatory and proangiogenic, metabolic inhibitors, bioactive gases, traditional Chinese medicine, cell-based therapy, etc.

**Table 3:** Therapeutic substances for reducing IRI.

Therapeutic substances category	Author, Year	Treatment drug	Ischemia method	Species	Number	Ischemia time	Reperfusion time	Skeleton muscle included
Group I Adenosine agoinst, endothelin antagonist, prostaglandin	Rowlands, 1999 (106)	prostaglandins (PG) E1, E2	Hindlimb IRI model	SD rats	82	Warm 6h	4h	Yes
	Luyt, Charles-Edouard, 2000 (107)	mixed ETA/B receptor antagonist, LU 135252	Hindlimb IRI model	Lewis rats	33	N/A	5h, 5d,14d	Yes
	Herbert, K. J, 2001 (108)	Bosentan	Hindlimb IRI model	SD rats	47	Warm 120 min	90min,24h	Yes
	JanFräßdorf, 2006 (109)	Prostaglandin E1	Hindlimb IRI model	Rabbits	64	Warm 45 min and 3h	2h,3h	Yes
	Zheng Jingang, 2007 (110)	Edenosine A1, A2A, and A3 receptors	Hindlimb IRI model	C57BL6	32	Warm 90 min	24h	Yes
Group II Complement inhibitor	Claudia Duehrkop, 2013 (111)	C1-inh	Hindlimb IRI model	Wistar rats	25	Warm 3h	24h	Yes
	C.Anton Fries, 2016 (83)	C1-inh	Free musculocutaneous flap model	Swine	12	Cold 3h	1d, 2d,7d,14d	Yes
	ShengyeZhang, 2018 (112)	C1-inh	Hindlimb IRI model	Wistar rats	28	Warm 2h	24h	Yes
	Inmaculada Masa, 2021 (113)	C1-inh	Superficial caudal epigastric skin flaps	Wistar rats	50	Warm 8h	7d	No
Group III Antioxidant	CengizBolcal, 2007 (114)	N-acetylcysteine, β-glucan, and coenzyme Q10	Hindlimb IRI model	New Zealand white rabbits	44	Warm 1h	3h	Yes
	Bradley D Medling, 2010 (115)	Vitamin E	Gracilis Muscle Flap Model	Wistar rats	12	Warm 4h	24h	Yes
	GuldenAvci, 2012 (116)	Curcumin	Hindlimb IRI model	Wistar rats	40	Warm 4h	2h	Yes
	Gan Muneuchi, 2013 (117)	D-allose	Abdominal skin island flap	Wistar rats	110	Warm 8h	8h	No
	Xu Dong, 2014 (118)	Dexmedetomidine	Hindlimb IRI model	Wistar rats	40	Warm 4h	2h	Yes
	Yin, Zhuming, 2016 (119)	Recombinant human thioredoxin-1	Dorsal lateral thoracic artery pedicled island skin flaps	CD-1	98	Warm 2h, 4h, 6h, 8h, 10h, and 12 h	24h	No
				mice				
	MircaferSeyid, 2021 (120)	Ceruloplasmin	Epigastric island flaps	SD rats	32	Warm 6h	24h	No
Group IV Anti-apoptosis	Kexin Song,2015 (121)	Methane-rich saline	Abdominal skin flap	SD rats	N/A	Warm 6h	72h	No
	Yedong Cheng, 2016 (122)	Pterostilbene	Hindlimb IRI model	SD rats	N/A	Warm 4h	4h	Yes
	Dawei Xin,2020 (123)	LXA4	Abdominal skin flap	Wister rats	54	Warm 8h	12h,24h,48h	No
Group V Anti-inflammatory angiogenesis	Elizabeth W Zhang,2015 (124)	Activated protein C	Gracilis muscle flap	SD rats	60	Warm 4h	1h,4h,18h,24h	Yes
	Dong Kyun Rah,2017 (125)	Platelet-Rich Plasma	Lateral thoracic artery island flaps	C57BL	30	Warm 4h	1d, 3d, 5d, 7d, 10d	No
	Sun-Young Nam,2018 (126)	NecroX-5	Abdominal skin flap	SD rats	20	Warm 7h	24h	No
Group VI Reduce metabolic	Henderson, Peter W, 2010 (127)	Hydrogen Sulfide	Hindlimb IRI model	C57BL6	42	Warm 3h	3h	Yes
Group VII Traditional chinese medicine	GangZhao, 2018 (128)	Irisin	Dorsal island skin flap	SD rats	48	Warm 6h	7d	No
	Gang Chen, 2018 (129)	luteolin	Abdominal skin flap	SD rats	18	Warm 4h	7d	No
	Huiwen Ren, 2018 (130)	Ganoderma lucidum Polysaccharide Peptide	Dorsal lateral thoracic artery pedicled island skin flaps	CD-1 mice	80	Warm 4h	24h,7d	No
	Yijia Xiang, 2018 (131)	Salvianolic acid	Hindlimb IRI model	SD rats	60	Warm 6h	24h	Yes
	YanZhao, 2019 (132)	epigallocatechin gallate	Hindlimb IRI model	SD rats	30	Warm 4h	6h	Yes
Group VIII Bioactive gases	Joon Pio Hong, 2003 (133)	Hyperbaric Oxygen	Abdominal skin flap	SD rats	100	Warm 3h	24h	No
	Aurelia Bihari, 2017 (134)	Carbon monoxide-releasing molecules	Hindlimb IRI model	Wistar rats	14	Warm 2h	1.5h	Yes
	Cagdas Elsurer, 2018 (135)	Ozone	Pectoralis muscle flap	Wistar rats	28	Warm 3h	7d	Yes
	Hao Cui, 2020 (136)	Nitric oxide (NO)	Rectangular island flap	Wistar rats	24	Warm 10h	12h	No
	Jian Tong, 2021 (137)	Hydrogen Gas	Hindlimb IRI model	C57BL/6	24	Warm 3h	4h	Yes
Group IX Cell based treatment or Mitochondrial transplantation	David W Hammers, 2015 (138)	Anti-inflammatory macrophages	Hindlimb IRI model	C57BL/6	21	Warm 2h	3d, 5d	Yes
	Alberto Ballestín, 2018 (139)	Adipose-Derived Stem Cells	Superficial caudal epigastric skin flaps	Wistar rats	28	Warm 8h	7d	No
	Yun Bai, 2018 (140)	Adipose mesenchymal stem cell-derived exosomes	Superficial inferior epigastric vessels	SD rats	18	Warm 6h	5d	No
	Arzoo Orfany, 2020 (141)	Mitochondrial transplantation	Hindlimb IRI model	C57BL/6	48	Warm 2h	24h	Yes

N/A, not available.

Despite the aforementioned therapeutic substances, the maneuver of postconditioning or remote postconditioning are effective therapies targeting IRI (142–145). Importantly, these strategies are simple, safe, and at least relatively harmless. Although the clinical trials of ischemic preconditioning or remote ischemic preconditioning have demonstrated favorable results in cardiac, hepatic, and pulmonary surgery, large, randomized, multi-center trials are required to verify the efficacy of these interventions in human skeletal muscle and skin. Recently, cutting-edge techniques have shown promising results, especially in muscle tissue preservation. The following paragraph describes these exciting methods.

## Cryopreservation

Cryopreservation aims to slow the deterioration of graft tissue by reducing the rate of metabolism (146–149). This requires freezing of the graft to temperatures below 0°Cand offers the possibility of storage for many weeks. To preserve tissue viability by cryopreservation, careful control of the rate of cooling is necessary, as well as the addition of cryoprotectants to prevent intracellular ice crystal formation (150–152). Several studies have described the applicability of preserving single-cell systems, blood vessels, cutaneous tissues, bones, and nervous tissues by cryopreservation (153–155). In 2008, Rinker et al. (156) preserved rat epigastric flaps at −140°C for 2 weeks. The authors then performed isotransplantation using the flaps, which remained viable for up to 60 days, maintaining normal pigmentation and hair growth, and showing no histological signs of inflammation or necrosis. Arav et al. (157, 158) performed the first directional freezing and vitrification to preserve a syngeneic heterotopic rat hindlimb for 7 days. They demonstrated that myocytes, blood vessels, and skin layers of the hindlimb remained histologically viable 3 days after transplantation. Studies on the effects of cryopreservation on human VCA grafts are currently lacking. Although long-term VCA graft storage is possible with cryopreservation, it is still challenging to establish a standard preservation guideline because different tissues respond differently to freezing, thawing, and cryoprotectants (159).

## Machine perfusion

The aim of machine perfusion is to preserve organ viability by supplying oxygen and nutrients and removing metabolic by-products (160–163). This way, grafts are preserved extracorporeally for extended periods, thereby significantly increasing their geographic accessibility (164–166). Grafts can be preserved under a variety of perfusion temperatures (167); these include hypothermic (0°C–12°C), mid-thermic (13°C–24°C), sub-normothermic (25°C–34°C), and normothermic (35°C–38°C) conditions. Studies utilizing small and large animal VCA models have shown that machine perfusion can effectively preserve transplant tissue for up to 24 hours (**Table 4**) (173, 187–191). Human limbs were preserved by Werner et al. (181) for 24 h using plasma-based sub-normothermic machine perfusion. After being preserved for 24 hours, the grafts were still functional and continued to respond to neuromuscular electrostimulation while exhibiting no evidence of myocyte damage.

**Table 4:** Machine perfusion in VCA.

Year	Author	Species	Model	N	Perfusion solution	Perfusion time	Perfusion temperature	Oxygenation	Outcomes
2022	Rezaei, M. et al (168)	Human	Upper extremities	20	Oxygenated red blood cell-based solution	41.6 ± 9.4hr	Normothermic (38°C)	Yes	MP overcome the limitations of SCS extending preservation times, enabling limb quality assessment, and allowing limb reconditioning before transplantation.
2022	Goutard M. et al (169)	Rat	Hindlimb	60	Modified Steen solution	3hr	Mid-thermic (21°C)	Yes	The use of MP for vascularized composite allografts could extend the preservation time and limit cold ischemia induce injury.
2022	Figueroa, B. A.et al (170)	Swine	Forelimb	24	Polymerized HBOC-201	22.5 ± 1.7hr	Normothermic (38°C)	Yes	MP with HBOC-201 could support isolated limb physiology, metabolism, and function
2022	Burlage, LC. et al (171)	Rat	Hindlimb	74	Acellular oxygen carrier HBOC-201	6hr	Mid-thermic (21°C)	Yes	Six hours MP using an acellular oxygen carrier HBOC-201 results in superior tissue preservation compared to SCS.
2021	Kruit, A. S. et al (172)	Swine	Forelimb	24	UW solution	16hr	Hypothermic (8°C-10°C)	No	*In-vivo* muscle contraction was well preserved after 18 h machine perfusion compared to short SCS,
2021	Amin, K. R.et al (173)	Swine	Forelimb	35	Matched blood	6hr	Normothermic (38°C); Subnormothermic (28°C); Hypothermic (10°C).	Yes	MP resulted in superior graft preservation and less reperfusion injury compared with the SCS.
2020	Said, S. A.et al (174)	Swine	Forelimb	3	HBOC-201	21.3 ± 2.1hr	Normothermic (39.8°C)	Yes	MP could preserve muscle contractility and mitochondrial structure compared to SCS
2020	Haug, V. et al (175)	Human	Upper extremities	6	Steen solution	24hr	Hypothermic (10°C)	Yes	MP with an oxygenated acellular Steen solution can extend the extracorporeal preservation time compared to SCS
2020	Haug, V. et al (176)	Swine	Forelimb	10	Dextran-enriched Phoxilium, Steen, or Phoxilium	12hr	Hypothermic (10°C or 4°C)	Yes	MP has been shown to be a promising alternative to (SCS for preservation of vascularized composite allotransplantation
2020	Fahradyan, V. et al (177)	Swine	Forelimb	10	Colloid solution containing washed RBCs	12-44hr	Normothermic (38°C)	Yes	Extended normothermic MP is a feasible option for preservation of amputated limbs.
2019	Krezdorn, N. et al (178)	Swine	Forelimb	8	Modified STEEN Solution	24hr	Hypothermic (8°C)	Yes	MP may reduce muscle damage and systemic reactions to limb replantation compared to SCS.
2019	Gok, E. et al (179)	Rat	Hindlimb	20	Swine hemoglobin and STEEN Solution	6hr	Near-normothermic (30°C-35°C)	Yes	Rat hindlimbs were viable after 6 hours of MP
2018	Krezdorn, N. et al (180)	Swine	Forelimb	8	Perfadex solution	2hr or 12hr	Hypothermic (10°C)	No	Ex vivo perfusion for up to 12 h is a viable alternative for preservation of vascularized composite tissues.
2017	Werner, N. L. et al (181)	Human	Upper extremities	5	Plasma-based with a hemoglobin	24hr	Near-normothermic (30-33°C)	Yes	Human limb allografts appeared viable after 24 hours of MP
2017	Kueckelhaus, M. et al (182)	Swine	Forelimb	7	Acellular Perfadex solution	12hr	Hypothermic (10°C)	Yes	MP could also be applied to the field of transplantation, expanding the potential pool of viable donor vascularized composite allografts.
2017	Duraes, E. F. R. et al (183)	Swine	Forelimb	18	Colloid solution containing red blood cells	12hr	Normothermic (39°C)	Yes	Ex-situ normothermic limb perfusion preserves limb physiology and function for at least 12 hours.
2016	Ozer, K. et al (184)	Swine	Forelimb	20	Autologous blood	24hr	Subnormothermic (27°C–32°C)	Yes	Successful prolongation of limb survival using MP provides with more time for revascularization of an extremity.
2015	Ozer, K. et al (185)^l^	Swine	Forelimb	14	Autologous blood	12hr	Subnormothermic (27°C–32°C)	Yes	MP could extend the narrow time frame for revascularization of procured extremities in limb transplantation.
2015	Araki J et al (186)	Rat	Hindlimb	15	ETK solution or HbV	6hr	Subnormothermic (22°C–27°C)	Yes	Oxygenic preservation is effective for rat ischemic limbs, suggesting that this method may be useful for other replantation and transplantation surgeries

HBOC, hemoglobin-based oxygen carrier; MP, machine perfusion; SCS, static cold storage; hr, hours.

Although recent studies have demonstrated the capability of machine perfusion in preserving graft tissue for an extended period (171, 192), some challenges still exist, such as (1) a paucity of studies utilizing allografts, (2) the absence of long-term follow-up data, and (3) lack of consensus on ideal temperature or perfusate for use in clinical settings. With the development of science and technology, machine perfusion combined with cryopreservation, CRISPR/Cas 9, stem cell therapy, siRNA, etc, to achieve *in vitro* editing of donor organs and modify the immunogenicity of donors, which can reduce IRI and immune rejection of the graft, and help the long-term survival of the graft (193–197).

## Summary

Our retrospective review found that IRI not only causes tissue damage but also increases acute and chronic rejection events, with consistent results in organ transplantation and VCA. However, VCA contains different tissue components, and muscle is a highly metabolically active tissue that is most susceptible to reperfusion injury. The traditional static preservation method has been unable to meet clinical needs. Long-term cold ischemia causes great muscle damage, which is extremely detrimental to the functional recovery of VCA.

Advances in science and technology, such as cryopreservation technology, machine perfusion technology, etc, have significantly prolonged the preservation time of VCA. These effects are significantly better than static preservation. However, these technologies still need to be further improved, and certain consensus should be reached to standardize their clinical usage.

## Future

Although VCA surgery is a life-improving, non-life-saving surgery, the ethics of surgery are still subject to academic controversy. The current focus of controversy is how to achieve a balance between patient cost and benefit. In order to improve the quality of life, patients need to take immunosuppressive drugs for a long time, and the side effects of these drugs greatly limit their clinical application. Recently, with the continuous deepening of basic research, the immune tolerance program of VCA has been successfully established in mice (198–201). But there are still many hurdles in translating it into large animals, even primates (202). IRI is one of the important factors that threaten the immune tolerance of VCA. In addition, improving the IRI could break geographic boundaries, expand the donor pool, increase organ utilization, and achieve better MHC-matching. At present, there are still few studies on IRI, and the pathophysiological mechanism of its tissue injury still needs to be further studied.

## Author contributions

JH wrote the article and made the figure. UK, LQ, PW and JT proofread the manuscript. JT reviewed the article. All authors contributed to the article and approved the submitted version.

## Conflict of interest

The authors declare that the research was conducted in the absence of any commercial or financial relationships that could be construed as a potential conflict of interest.

## Publisher’s note

All claims expressed in this article are solely those of the authors and do not necessarily represent those of their affiliated organizations, or those of the publisher, the editors and the reviewers. Any product that may be evaluated in this article, or claim that may be made by its manufacturer, is not guaranteed or endorsed by the publisher.
